# "hCG priming" effect in controlled ovarian stimulation through a long protocol

**DOI:** 10.1186/1477-7827-7-91

**Published:** 2009-08-31

**Authors:** Panagiotis Beretsos, George A Partsinevelos, Eleni Arabatzi, Peter Drakakis, Depy Mavrogianni, Elli Anagnostou, Kostas Stefanidis, Aris Antsaklis, Dimitris Loutradis

**Affiliations:** 1 1st Department of Obstetrics and Gynaecology, Division of Human Reproduction, IVF Unit, Athens University Medical School, "Alexandra" Hospital, Athens, Greece

## Abstract

**Background:**

Recently, it has been demonstrated that, in patients down-regulated by GnRH analogues (GnRHa), a short-term pre-treatment with recombinant LH (rLH), prior to recombinant FSH (rFSH) administration, increases the number of small antral follicle prior to FSH stimulation and the yield of normally fertilized embryos. However, no data exist in the literature regarding the potential beneficial effect of "hCG priming" in controlled ovarian hyperstimulation (COH) through a long GnRH-a protocol, which binds the same receptor (LH/hCGR), though it is a much more potent compared to LH. The primary aims of this study were to assess the effect of short-term pre-rFSH administration of hCG in women entering an ICSI treatment cycle on follicular development, quality of oocytes and early embryo development. The secondary endpoints were to record the effects on endometrial quality and pregnancy rate.

**Methods:**

Patients with a history of at least one previous unsuccessful ICSI cycle were randomly assigned into two groups to receive treatment with either a long protocol with rFSH (control group) or a long protocol with rFSH and pre-treatment with hCG (hCG group). In particular, in the latter group, a fixed 7 days course of 200 IU/day hCG was administered as soon as pituitary desensitization was confirmed.

**Results:**

The mean number of oocytes retrieved was not significantly different between the two treatment groups, although the percentage of mature oocytes tended to be higher but not significantly different in hCG-treated patients. The percentage of patients with more than one grade 3 embryos was higher in the pre-treatment group, which also showed a higher pregnancy rate.

**Conclusion:**

All the above clinical observations, in conjunction with previous data, suggest a point towards a beneficial "hCG priming" effect in controlled ovarian hyperstimulation through a long GnRH-a down-regulation protocol, particularly in patients with previous ART failures.

## Background

Ovarian response to gonadotrophins varies considerably among women. The importance of this differential response in women with previous ART (IVF/ICSI) failures has prompted researchers to investigate and determine the factors that are implicated [1].

Though, follicular development up to the preantral stage is feasible in the absence of LH, an essential role for this gonadotrophin for antral formation as well as further growth and differentiation has been uniformly recognized. LH plays a key role in both oocyte and follicular cells development through modification of the steroid and protein micro- and macroenvironment [2,3]. These physiologic changes have a prominent role in oocyte, maturation, the process of ovulation, and subsequent fertilization and implantation [4].

It is well known that LH acts synergistically with FSH in the process of follicular growth: FSH plays a crucial role in recruitment, selection and dominance, while LH contributes to dominance maturation and ovulation [2,5]. Moreover, studies in non-human primates have shown that LH may act by increasing intra-ovarian androgens, which in turn promote FSH responsive granulosa cell function [6]. Recently, it has been demonstrated that, in patients down-regulated by GnRH analogues (GnRHa), a short-term pre-treatment with recombinant LH (rLH), prior to recombinant FSH (rFSH) administration, increases the number of small antral follicles prior to FSH stimulation and the yield of normally fertilized (2PN) embryos[7]. In addition, rLH pre-treatment may have a modest impact on subsequent ovarian responsiveness to FSH. LH activity, administered as a single dose of hCG in combination with aromatase inhibitor in early-follicular-phase GnRH-antagonist protocol has been shown to result in androgen priming and subsequent increase in the number of good quality embryos [3]. However, no data exist in the literature regarding the potential beneficial effect of "hCG priming" in controlled ovarian hyperstimulation (COH) through a long GnRH-a protocol, given that hCG occupies the same receptor (LH/hCGR) though it is much more potent than LH.

The ideal LH activity, administered as hMG, rLH or hCG in ART procedures, has not been determined yet. Serum LH levels of less than 1.5 IU/L have been proven insufficient to maintain aromatase activity and E_2 _production [8]. Low peri-ovulatory levels (<3 IU/L) in patients undergoing IVF are associated with impaired fertilization and increased early pregnancy loss [9,10]. On the other hand, small doses of LH administered in early follicular phase during ovarian stimulation in IVF-ET cycles have a beneficial effect in the quality of oocytes, a fact of utmost importance, especially in cases where few embryos are available for transfer [11]. Actually, in poor responders, early LH administration during COH may have a beneficial effect on the maturity and fertilizability of oocytes, as well as the number of transferable embryos [5,12,13]. However, combined LH and FSH activity administration did not yield increased pregnancy rates [14,15]. In a recent meta-analysis of several randomized controlled trials, investigating the effect of rFSH alone or in combination with rLH in IVF/ICSI cycles, no evidence of a statistical difference regarding pregnancy outcome in patients where rLH was used was observed [16].

In common practice, human chorionic gonadotrophin (hCG) is used as a substitute for the mid-cycle LH surge, due to the degree of homology between the two hormones [17]. hCG has long been associated with the initiation and maintenance of pregnancy. A potential role for hCG has been suggested lately in the processes associated with infertility [18-22]. Although the two molecules have the same natural function, causing luteinization and supporting lutein cells, hCG has a slower plasma metabolic clearance, which consists of a rapid phase in the first 5-9 h following IM administration and a slower phase in the first 1-1.3 days after administration. Both LH and hCG are complex heterodimeric glycoproteins with different molecular weights (30 KD and 40 KD respectively). Difference in their carbohydrate moiety possibly explains different affinity to the LH/hCG receptor and therefore differentiated function [23,24]. In the absence of FSH, low-dose hCG can support development and maturation of larger ovarian follicles (≥15 mm in diameter) that have acquired granulosa cell LH/hCG receptors and hasten the demise of smaller follicles lacking these receptors thus being dependent on FSH stimulation [19,25,26]. Inasmuch as the development of small preovulatory follicles is associated with increasing rates of ovarian hyperstimulation syndrome, the addition of low dose hCG might provide effective and safer ovulation induction regimens [19,25,27]. Moreover, hCG seems to be capable of positively affecting uterine receptivity by enhancing endometrial quality and stromal fibroblast function. In particular, hCG may improve intrauterine environment and extend implantation window thus increasing pregnancy rate through its actions on insulin-like growth factor binding protein-1 (IGFBP-1) and vascular endothelial growth factor (VEGF) [11,25,28].

The primary aims of this study were to assess the effect of short-term pre-rFSH administration of hCG in women entering an ICSI treatment cycle on follicular development, quality of oocytes and early embryo development. The secondary endpoints were to record the effects on endometrial quality and pregnancy rate.

## Methods

### Patient population

Fifty patients with a history of at least one previous unsuccessful ICSI cycle were enrolled into this prospective randomized pilot study. The inclusion criteria were pre-menopausal woman, 25-40 years of age and a normal hormonal profile (according to WHO guidelines). None of the patients had received ovulation induction or any other hormone treatment for at least three months preceeding the study.

The patients were randomly assigned into two groups: Group 1 underwent a long protocol with rFSH (Control Group) and Group 2 received a long protocol with rFSH and pre-treatment with hCG (hCG Group). To perform randomization a random number generator  was used. For each patient, a random number between 1 and 100,000 was generated and the patient was allocated to the corresponding group (Group 1 for odd numbers and Group 2 for even numbers).

The demographic data (age, duration of infertility, previous attempts, weight and height) for all patients was collected and their BMI was calculated. Cycle day 2 FSH, LH and PRL were measured prior to the ICSI cycle within the previous six months.

### Ovulation induction

The protocol was approved by our Ethics Committee and an informed consent was provided by all participants, according to the Helsinki Declaration. The stimulation protocol was briefly as follows: on day 21 of the previous cycle, a baseline ultrasound scan was performed and buserelin intranasal spray (Superfact; Hoechst, Frankfurt, Germany) was commenced at a dose of 100 μg five times daily (every 4 h, omitting the 3.00 a.m. dose) for 14 days. In all of the patients, the extent of ovarian suppression was evaluated by both ultrasound scan (absence of ovarian activity, ovarian cyst formation and endometrial proliferation) and serum E_2 _levels (≤ 40 pg/ml) before starting exogenous gonadotrophin administration. If the above criteria were not met, downregulation was extended for a further week. Group 2 patients were pre-treated for seven days with 200 IU/day of hCG (Pregnyl; N.V. Organon, Oss, Netherlands) when ovarian suppression had occurred. Stimulation was commenced with rFSH (Gonal-F; Serono, Geneva, Switzerland) at a fixed dose of 200 IU daily in both groups.

Plasma E_2 _levels were measured five days after starting rFSH and then daily from day 8. The first scan was performed on day 9 and subsequent scans were performed every day.

The dose of rFSH was adjusted according to ovarian response after 5 days of rFSH administration. GnRHa administration was continued until hCG administration for triggering ovulation. In particular, 10,000 IU were given intramuscularly when the mean diameter of at least two leading follicles was >18 mm and serum E_2 _was rising. The interval between the last gonadotrophin injection and hCG administration was no more than 24 h. Thirty-five to 36 h after hCG administration, ovum retrieval was performed by transvaginal echo-guided ovarian puncture. After stripping, oocytes were assessed for their maturation and only oocytes having resumed their first meiotic division reaching metaphase II were used for ICSI.

The embryos were graded according to their morphologic appearance on a scale from 3 (the best) to 1 under a light microscope on the day of transfer [29]. Two to three embryos were transferred according to embryo quality assessment. Luteal phase was supported with 2,500 IU of hCG injected on the days of embryo transfer and day 4 after replacement. Serum hCG was measured 14 days after oocyte retrieval. Clinical pregnancy was confirmed by a gestational sac with fetal heartbeat movement seen on transvaginal ultrasound scan two weeks later.

Assessment of follicular growth and endometrial thickness by ultrasound scan as well as oocyte collection were performed by the same fertility specialist being unaware of the study group in which the patient was assigned. Similarly, evaluation of oocyte maturation, ICSI procedure and embryo quality assessment were performed by a unique embryologist who was blinded too.

### Study endpoints

The primary aim of this study was to assess the effect of short-term pre-rFSH administration of hCG in women entering an ICSI treatment cycle on follicular development, quality of oocytes and early embryo development. To this end, ovarian ultrasound profile, serum estradiol level on day 5 of rFSH administration, serum E_2 _level and E_2 _level per follicle on the day of triggering ovulation, number of oocytes collected and oocyte maturation rate were recorded. The secondary endpoints were the measurement of the effects on endometrial quality (endometrial thickness>8 mm) and on ICSI outcome expressed by fertilizationand pregnancy rates.

### Statistical evaluation

ANOVA models were used to detect a group or treatment effect. Tests of normality and equality of variance of the residuals were applied for validation of the model. Estimates, P values and 95% confidence intervals (CIs) of the difference between groups and/or treatments were computed. For efficacy parameters for which several measurements were obtained on the same patient (i.e. oocyte nuclear maturity, grading of embryos), a generalized mixed linear model was applied. For proportions (i.e. pregnancies), a logistic regression model was used. Log odds ratios, P values and 95% CIs of the log of the odds ratio between groups and treatments were computed. Laboratory and safety measures, unless otherwise noted, were analyzed using the same statistical tests.

## Results

All 50 patients that were initially included in this pilot study were randomized into two groups: Group 1 (long protocol with rFSH) consisted of 28 patients and Group 2 (long protocol with rFSH + 7 days hCG) consisted of 22 patients. Four patients (one from Group 1 and three from Group 2) dropped-out of the study before ovulation induction for personal reasons. All patients were pre-menopausal, aged 25-39 years (median age: 33 years) and had a normal hormonal profile. The indication for ICSI was male infertility, but in a minor subset of the patients, equally distributed between the two study groups, one or more of the following co-existing causes of infertility were present: tubal factor, anovulation and endometriosis. The patients' demographic characteristics and hormonal profiles were similar in both groups (Table 1).

**Table 1:** Patients' demographic mean characteristics and hormonal profiles based on the administered treatment.

	Group 1 (r-FSH) n = 27	Group 2 (r-FSH+hCG) n = 19
Age (years)	33 ± 4	34 ± 4
Duration of infertility (years)	6 ± 4	6 ± 2
Previous attempts	1 ± 0	1 ± 1
Weight (Kgs)	59 ± 13	64 ± 10
Height (cm)	162 ± 8	168 ± 5
BMI	22.5 ± 5.2	23 ± 4.4
FSH (mlU/ml)	7.5 ± 3.2	6.7 ± 1.2
LH (mlU/ml)	5.2 ± 1.8	4.6 ± 3.1
PRL (mg/ml)	13.9 ± 5.8	14.4 ± 5.3

All the patients had menstruated before starting either FSH or hCG administration. Ovarian response in both groups is presented in Table 2. The duration of ovarian stimulation, as well as the total dose of gonadotrophins, were similar in both groups. Patients in the two groups did not show any significant difference in their E_2 _levels on day 5 of rFSH administration (259.3 ± 175.8 pg/ml in Group 1 vs. 305 ± 214.4 pg/ml in Group 2 p > 0.05), but those in Group 2 showed higher serum E_2 _levels on the day of hCG administration (ovulation induction) (1,643.5 ± 800.2 pg/ml in Group 1 vs. 2,125 ± 1,190.2 pg/ml in Group 2 p < 0.05). Serum E_2 _levels per follicle on the day of hCG administration tended to be higher in Group 2, but not significantly different (200 ± 98.9 pg/ml in Group 1 vs. 139 ± 116.9 pg/ml in Group 2 p > 0.05).

**Table 2:** Patients' ovarian response based on the administered treatment (mean values, * = p < 0.05).

	Group 1 (r-FSH) n = 27	Group 2 (r-FSH+hCG) n = 19
E_2 _on day 5 of FSH admin.	259.3 ± 175.8	220 ± 214.4
E_2 _on day of hCG admin.	1643.5 ± 800.2	2125* ± 1190
E_2_/follicle on day of hCG admin.	200 ± 98.9	239 ± 116.9
No. of follicles	8 ± 3	10 ± 3
No. of oocytes	7 ± 3	8 ± 2
Mature oocytes (%)	66.7 ± 17	78.9 ± 18
Fertilized oocytes (%)	87.5 ± 12.5	85 ± 15
Embryo quality (%)	47.6	85.3*
Endometrial quality (%)	46.4	61.3*
Pregnancy Rate (%)	31.8	46.2*

Embryo quality is defined as patients with more than one Grade 3 embryos; Endometrial quality is defined as patients with endometrial thickness ≥ 8 mm.

The mean number of oocytes retrieved per patient was comparable between the two groups (7 ± 3 in Group 1 vs. 8 ± 2 in Group 2, p > 0.05), although the percentage of mature oocytes per retrieval per patient tended to be higher in Group 2 (66.7% ± 17% vs. 78.9% ± 18% respectively, p > 0.05). The number of fertilizable oocytes obtained was not significantly different between the two groups (5 ± 2 vs. 6 ± 2 respectively, p > 0.05), such as the fertilization rate (87.5% ± 12.5% vs. 85% ± 15% respectively, p > 0.05). In addition, the percentage of women that had more than one grade 3 embryos in Group 1 was significantly lower compared to that in Group 2 (women with grade 3 embryos: 47.6% in Group 1 vs. 85.3% in Group 2 p < 0.05). Similarly, patients that were included in the hCG pre-treatment group (Group 2) showed a significantly better endrometrial quality assessed by endometrial proliferation on the ultrasound scan (endometrial thickness > 8 mm) during embryo transfer (46.4% in Group 1 vs. 61.3% in Group 2, p < 0.05). Finally, pregnancy rate (PR) in Group 1 was 31.8%, compared to 46.2% in Group 2 (p < 0.05).

## Discussion

The identical alpha subunit and the significant sequence homology of beta subunit between LH and hCG, results in the exertion of both hormones' activity through the same receptor, LH/hCGR. However, hCG has a longer half-life and is much more potent than LH [30]. The long-acting profile of hCG renders this compound more attractive in terms of LH activity, as it can provide more prolonged and stable stimulation of LH/hCG receptors than rLH in between daily hormone administrations [19]. Although LH activity in COH is feasible with the administration of rLH, hMG or hCG, relatively few data exist regarding the use of hCG.

Daily doses of 50-200 IU of hCG have been used to supplement FSH in COH or even substitute FSH in the late follicular phase, so far [19-22]. A single dose of 1250 IU was implemented in a GnRH-antagonist protocol in combination with aromatase inhibitor in early-follicular-phase [21]. A fixed 7 days pre-treatment with 300 IU rLH in a long GnRH-a protocol was conducted recently [7]. We opted 200 IU hCG daily dose, the maximum dose ever used in relevant studies, because we designed to start hCG as soon as pituitary desensitization had occurred prior and not concurrently with rFSH administration. Given that LH/hCG receptors are present only on theca and not granulosa cells by that time, we did not decide on a higher dose to avoid possible side effects of excessive androgen production, which may compromise follicular potential. Concerning the timing of hCG administration, we assumed that pre-treatment instead of combining hCG with rFSH would have a better effect on follicular responsiveness to FSH as discussed below.

It is well known that follicular development is highly dependent on pituitary secretion of FSH and LH. These hormones are essential for normal follicular E_2 _production, an action presented in the literature as the two-cell two-gonadotrophin theory [31,32]. In fact, LH induces androgen production by the theca cells, whereas FSH promotes aromatase enzyme expression and thus the utility of androgens as a substrate for estrogen biosynthesis. Moreover, it has been shown that androgens themselves promote aromatase activity [2]. Thus, it could be speculated that LH-mediated androgen production increases follicular responsiveness to FSH [7]. In this context, Durnerin *et al *found that a fixed 7 days treatment with rLH prior to rFSH administration in a long GnRH-a protocol increased the number of small antral follicles prior to FSH stimulation and yielded an increased number of normally fertilized embryos [7]. In line with these data, our study showed that the percentage of patients with more than one grade 3 embryos was higher in the hCG pre-treatment group. Furthermore, that group had also a higher pregnancy rate. A recent study, in which hCG was administered as a single dose in combination with aromatase inhibitor in early-follicular-phase GnRH-antagonist protocol, resulted in increased number of good quality embryos. However, pregnancy rate did not differ between the study groups and implantation rate was significantly lower in the hCG group. Possible explanations for the different "hCG priming" effect on pregnancy rate in comparison to our results, and furthermore, the lower implantation rate observed in hCG-treated patients, could be the application of GnRH-antagonist instead of agonist protocol, which would have affected the quality of the endometrium [33] and aromatase inhibitor which is known to interfere in the process of steroidogenesis decreasing estrogen production and thus endometrial proliferation [34].

GnRH agonists are mainly characterized by their pituitary desensitization effect. However, they are also known to cause an early receptor activation leading to a flare up effect. This effect may be crucial for follicular recruitment, especially in the early stages of the maturation process. Several studies support this hypothesis, since discontinuing the GnRH agonist after five days of administration resulted to a recondite fall of LH levels in comparison to an unceasing administration [35]. In addition to the amount and duration of GnRH agonist treatment, a recent study has shown that ovarian response and pregnancy rates correlated to the route of administration [36]. In our study, intranasal buserelin resulted in significantly less depressed mid-follicular LH levels and increased pregnancy rates. This observation can reason for the variability in the treatment outcome of otherwise similar protocols that used s.c. administration. Furthermore, it may explain the differences in residual LH levels reported by previous studies [10,37-39].

Our study shows that hCG pre-treated women tended to have higher E_2 _levels on the day of hCG administration for triggering ovulation and resumption of meiotic division, which have been suggested to be related to better quality embryos and increased pregnancy rates. Actually, this was also the fact in our study. A possible explanation could be the direct induction of theca cells androgen production which is subsequently transformed to estrogen in granulosa cells through an increased aromatization rate.

We also found a beneficial effect of hCG pre-treatment on endometrial quality defined as endometrial thickness >8 mm, assessed by ultrasound scan on the day of egg collection. In fact, the thickness of the endometrium on the day of oocyte retrieval was statistically higher in the hCG group (>8 mm, 61.3% vs. 46.4 in Group 1, p < 0.05). This feature was related to a tendency for a higher pregnancy rate compared to the control group. Increased endometrial thickness could be attributed to enhanced estrogen production. However, Fillicori *et al. *have shown that hCG has the potential to improve uterine receptivity by enhancing endometrial quality and stromal fibroblast function [40]. In fact, through its mechanism that involves insulin-like growth factor binding protein-1 (IGF-1) and vascular endothelial growth factor (VEGF), hCG probably stimulates endometrial angiogenesis and extends the implantation window. This could explain the more efficient implantation rate in patients that received adjuvant treatment with hCG in our study. This observation suggested for the first time that pre-treatment with hCG may lead to an improved endometrial receptivity profile. It could be postulated that early hCG administration ameliorates the receptivity of the endometrium by enhancing endometrial environment and stromal fibroblast function [41,42].

Several studies support the implication of LH/hCG activity during early luteal phase in endometrial preparation for implantation [43,44]. On the other hand, comparably few data exist regarding the potential effects of hCG during early follicular phase on the functional maturation of endometrium. It is well known that under physiologic conditions, hCG is not present during this period. However, preovulatory endometrium is variably exposed to this hormone in all COH protocols that use HMG or purified gonadotrophins. The main exposure occurs at the end of the stimulation process, when the ovulation-triggering dose of hCG is administered.

Current evidence implies that simply raising the daily dose of rFSH only partially compensates for the ovaries which are resistant to gonadotrophins. On the other hand, soon after preantral-antral follicle transition, granulosa cells develop LH receptors, hence becoming sensitive to LH as well as hCG stimulation. Besides, LH or hCG-mediated theca cell androgen production has been suggested to increase follicular responsiveness to FSH. Thus, it could be hypothesized that granulosa cells resistant to rFSH stimulation might benefit from low dose hCG administration during the early follicular phase.

## Conclusion

In summary, all these clinical data suggest a point towards a beneficial "hCG priming" effect particularly in patients with previous IVF/ICSI failures. However, larger scale studies targeted in ovarian dysfunction patients are needed to elucidate a potential role for hCG pre-treatment in this poorly responding infertile subpopulation.

## Competing interests

The authors declare that they have no competing interests.

## Authors' contributions

DL and PB were responsible for designing and coordinating the study. EA, PD, EAN, GAP and KS were responsible for data collection. DL and PB were responsible for the statistical work and DL, DM, GAP and PB for writing of the manuscript. AA reviewed the article. All authors read and approved the final manuscript.
